# Kleefstra syndrome in Hungarian patients: additional symptoms besides the classic phenotype

**DOI:** 10.1186/s13039-016-0231-2

**Published:** 2016-02-25

**Authors:** Kinga Hadzsiev, Katalin Komlosi, Marta Czako, Balazs Duga, Renata Szalai, Andras Szabo, Etelka Postyeni, Titanilla Szabo, Gyorgy Kosztolanyi, Bela Melegh

**Affiliations:** Department of Medical Genetics, Clinical Center, University of Pecs, Szigeti 12, H-7624 Pecs, Hungary; Szentagothai Research Center, University of Pecs, Ifjusag 20, H-7624 Pecs, Hungary

**Keywords:** Kleefstra syndrome, 9q subtelomeric deletion syndrome, Epilepsy, Drug metabolism

## Abstract

**Background:**

Kleefstra syndrome is a rare genetic disorder, with core phenotypic features encompassing developmental delay/intellectual disability, characteristic facial features – brachy(micro)cephaly, unusual shaped eyebrows, flat face with hypertelorism, short nose with anteverted nostrils, thickened lower lip, carpmouth with macroglossia - and childhood hypotonia. Some additional symptoms are observed in different percentage of the patients. Epilepsy is common symptom as well. The underlying cause of the syndrome is a submicroscopic deletion in the chromosomal region 9q34.3 or disruption of the euchromatin histone methyl transferase 1.

**Case presentation:**

We describe two Hungarian Kleefstra syndrome patients, one with the classic phenotype of the syndrome, the diagnosis was confirmed by subtelomeric FISH. Meanwhile in our second patient beside the classic phenotype a new symptom – abnormal antiepileptic drug metabolic response – could be observed. Subtelomere FISH confirmed the 9q34.3 terminal deletion. Because of the abnormal drug metabolism in our second patient, we performed array CGH analysis as well searching for other rearrangements. Array CGH analysis indicated a large – 1.211 Mb -, deletion only in the 9q subtelomeric region with breakpoints ch9:139,641,471-140,852,911.

**Conclusions:**

This is the first report on Kleefstra syndrome in patients describing a classical and a complex phenotype involving altered drug metabolism.

## Background

Kleefstra syndrome (OMIM 610253) (KS), also known 9q subtelomeric deletion syndrome is a rare genetic disorder. The definite incidence is yet unknown, however, since the widespread application of subtelomeric FISH and later of array comparative genomic hybridization (aCGH) a number of new cases were diagnosed. In the phenotype of the syndrome developmental delay/intellectual disability, characteristic facial features – brachy(micro)cephaly, unusual shaped eyebrows, flat face with hypertelorism, short nose with anteverted nostrils, thickened lower lip, carp mouth with macroglossia - and childhood hypotonia are present in all of the patients [1]. Additional symptoms, as heart defects, microcephaly, genital/renal anomaly, recurrent infections, hearing impairment, tracheo/bronchomalacia are observed in different percentage of the patients. Epilepsy and psychiatric problems are important and common symptoms. While epilepsy is generally well controlled with standard medications, psychiatric abnormalities include apathy, aggressive periods, psychosis, autistic features, bipolar mood disorders and regression in daily function and cognitive abilities. There are also some other, less common symptoms observed, like micropenis, cryptorchidism and vesicouretheral reflux [1–4]. The underlying cause of the syndrome is, in the majority of the patients, a submicroscopic deletion in the chromosomal region 9q34.3 or disruption of the Euchromatin Histone Methyl Transferase 1 (Eu-HMTase1), which leads to haploinsufficiency of the *EHMT1* gene. In a study Kleefstra et al. found that the minimum critical region is 1.2 Mb and includes 14 genes on the long arm of chromosome 9, which could be responsible for the 9q subtelomeric deletion syndrome [5]. No genotype-phenotype correlation was observed so far with the size of the deletion or between patients with deletions and those with mutations. Meanwhile in her study Yatsenko et al. found that specific clinical endophenotypes are correlated with the extent of the deletion [6].

There are also some patients showing the core phenotypic features, but with phenotypic heterogeneity of KS in whom Kleefstra et al. identified de novo mutations in four epigenetic regulator genes, namely in *MBD5, MLL3, SMARCB1 and NR1I3* [7].

We describe KS in Hungarian patients for the first time. Of the two patients identified one showed the classic phenotype of the syndrome, a second patient presented with a new symptom beside the classic phenotype – namely an abnormal antiepileptic drug (AED) metabolic response.

## Case presentation

### Patients

The two hereafter outlined patients were referred to our clinic because of developmental delay and minor anomalies.

**Fig. 1:**
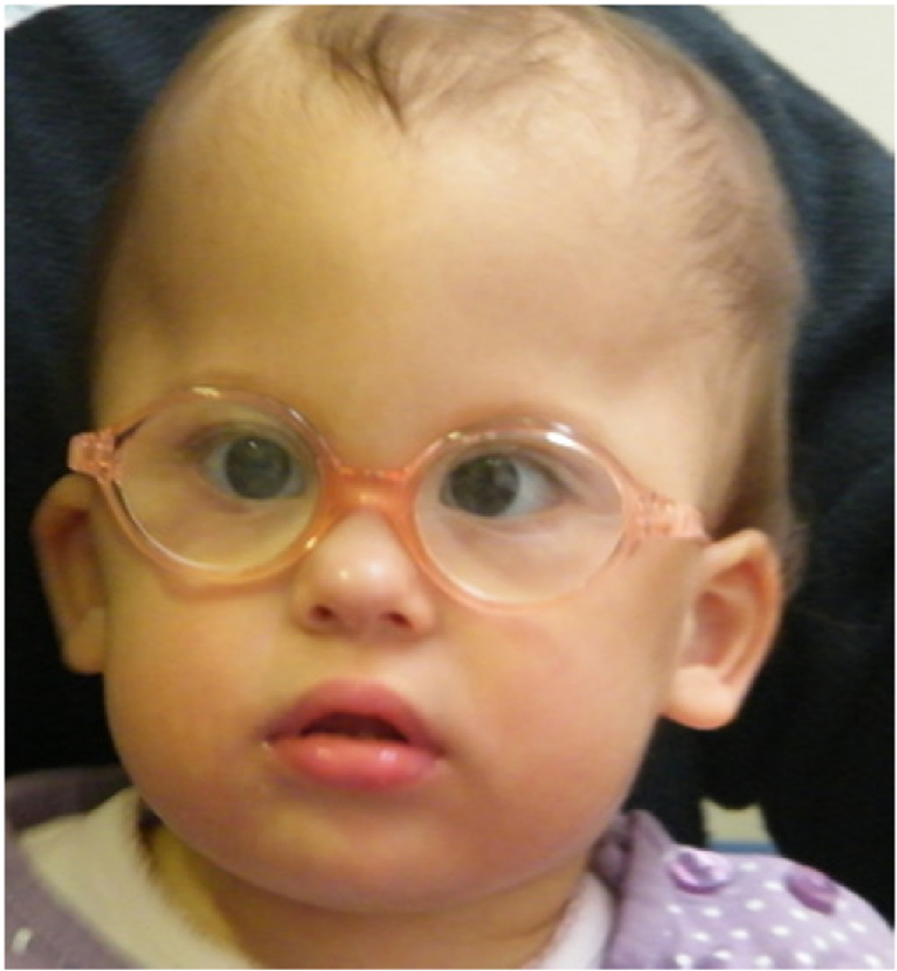
Characteristic features of Patient 1

**Patient 1**. is a 22 months old girl from the first pregnancy of a non-consanguineous healthy young Caucasian couple (father 30 and mother 26 years old). The only remarkable point in her family history is thyroid hypofunction in her mother, in the two sisters of her mother and in the maternal grandmother. Following the diagnosis of a hypoplastic aortic arch at the 26^th^ week of pregnancy the girl was delivered at 40 weeks of gestation with a birth weight of 2740 g (10–25 percentiles). An aortic stenosis and coarctation of the aortae was confirmed by cardiological examination on the first day of life. Her developmental milestones were delayed. She turned at 8 months, at 20 months she sat alone and tried to crawl, and her eye contact evolved around 8 months. Babbling started timely but stopped at 20 months. The objective audiometry showed bilateral hearing impairment. After adjustment of a hearing aid, considerable advance was not detected in her auditive attention. The first epileptic seizure developed at 22 months, which had an adequate therapeutic response to valproate treatment. Brain MRI detected symmetrical dilated liquor space with a consequent gracile hippocampus and subcortical ischemic lesions. The characteristic features are illustrated in Fig. 1.

She was referred to our institution because of dysmorphic features at the age of 8 months. At that examination her weight was 7850 g (50 percentile) height 68 cm (25–50 percentile) and OFC 42 cm (−1 SD), she had brachycephaly, flat face, midface hypoplasia, down-slanting palpebral fissure, convergent strabismus, short nose, high palate, tented lip and severe generalized hypotonia.

**Fig. 2:**
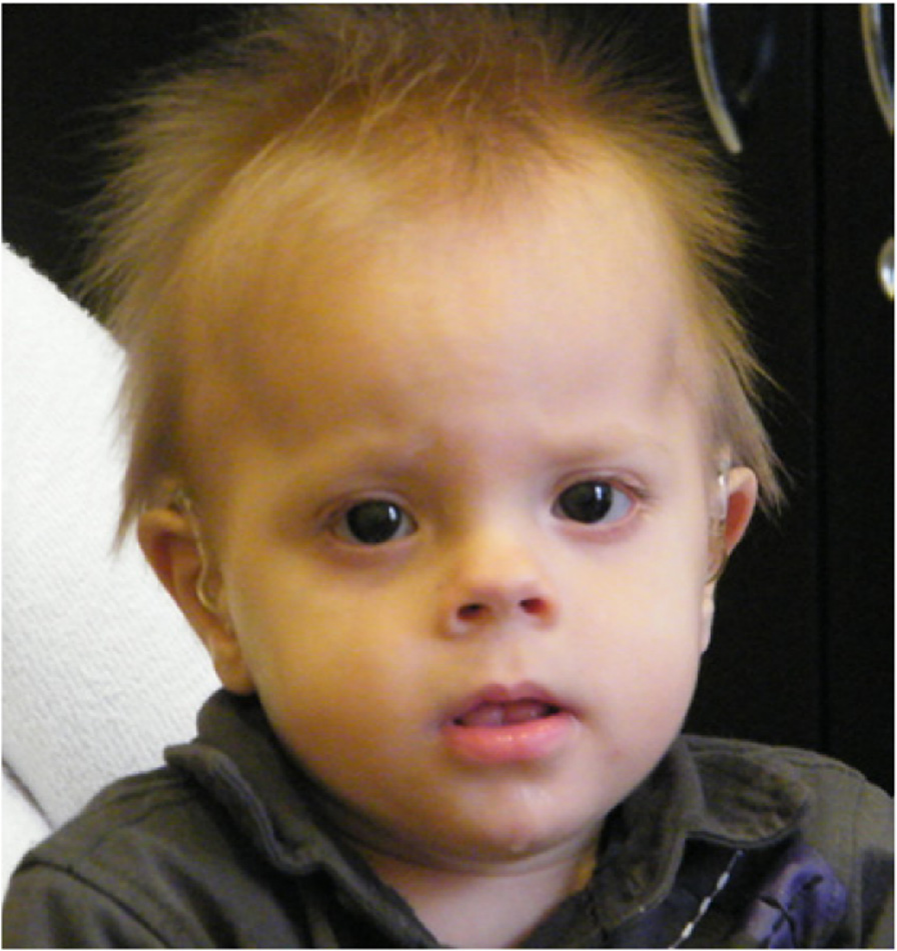
Patient 2 with representative phenotype

**Patient 2**. is a 30 month old boy, second child (G2P1) of a non-consanguineous healthy young couple (father is 34 and mother is 31 years old), his family history was unremarkable. Because of endometriosis hormone therapy was applied in the mother before pregnancy. In the fetus dysmaturity was observed from the 34th week of gestation. He was delivered at 38 weeks of gestation with a birth weight of 2600 g. His developmental milestones were delayed, he sat alone at 12 months, stood at 15 months but at 18 months he could not walk. Speech development was delayed as well, only bubbling was present at 18 months. The objective audiometry showed bilateral hearing impairment and in the first year of life he went through multiple pneumonias. The pulmonological examination revealed tracheomalacia in the background. His first epileptic seizure developed at 20 months of age, since then the seizures are therapy-resistant focal seizures. An abnormal AED metabolism was observed in the boy, namely minimal AED doses already cause a toxic drug blood level (at 0.03 mg/kg clonazepam 600 nmol/l blood level and at 11 mg/kg levetiracetam 330 nmol/l blood level occur). At 18 months of age as he was first examined in our institution his weight was 10 kg (<5 pc) height was 81 cm (<5 pc) and OFC was 45 cm (<−2 SD). Brachymicrocephaly, flat face, midface hypoplasia, hypertelorism, short nose, tented lip, thick lower lip, pointed chin, malformed ears and mild hypotonia were present. The characteristic features can be seen in Fig. 2.

### Results

**Patient 1**: GTG-banded chromosomes at the 450 (550)-band level showed 46, XX normal female karyotype and subtelomeric FISH confirmed the 9q34.3 terminal deletion. The size of the deletion (2.188 Mb) was established by array CGH: ch9:138,831,145–141,018,984.

**Patient 2**: GTG-banded chromosomes at the 450 (550)-band level were normal and subtelomeric FISH confirmed the 9q34.3 terminal deletion. Performed aCGH indicate a 1.211 Mb deletion in the 9q subtelomeric region with the breakpoints ch9:139,641,471–140,852,911.

## Conclusions

In the present work we describe two unrelated Hungarian children with a phenotype characteristic of Kleefstra syndrome. We made a comparison between the symptoms of our patients and of patients with 9q subtelomeric deletions presented by Kleefstra (Table 1) [8].

**Table 1:** Clinical features of the presented cases compared with patients with 9q subtelomeric deletion presented by Kleefstra et al.

Features	Kleefstra et al. [8]	Patient 1	Patient 2
Gender	…	-	+
MR	28/28 (100)	+	+
Obesity	5 (16)	-	-
Microcephaly	25 (83)	-	+
Brachycephaly	12 (33)	-	-
Flat face	5 (16)	+	+
Midface hypoplasia	16 (53)	+	+
Coarse facies	8 (27)	-	-
Hypertelorism	15 (50)	-	+
Synophrys	16 (53)	-	-
Down slant palpebral fissures	6 (20)	+	-
Up slant palpebral fissures	5 (16)	-	+
Arched eyebrows	8 (27)	-	-
Short nose	17 (57)	+	+
Anteverted nostrils	12 (33)	-	-
Carp mouth	23 (77)	+	+
Macroglossia	12 (33)	-	-
Natal teeth	1 (3)	-	-
Thick lower lip	5 (16)	-	+
Pointed chin	5 (16)	-	+
Malformed ears	12 (33)	-	+
Brachydactyly	5 (16)	-	-
Simian crease	10 (30)	-	-
Abnormal male genitals	10 (33)	-	
Cardiac anomaly	15 (50)	+	-
Anal atresia	2 (7)	-	-
Alopecia	3 (10)	-	-
Depigmentation	1 (3)	-	-
Renal cysts	2 (7)	-	-
Hydronephrosis	2 (7)	-	-
Behavioral problems	4 (9)	-	+
Sleep disturbances	3 (10)	-	-
Hearing loss	6 (20)	+	+
Hypotonia	15 (50)	+	+
Seizures	10 (30)	+	+

Initially, regarding Patient 1 with terminal deletion of 9q, she was merely diagnosed using conventional FISH method. Her facial features (brachycephaly, flat face, midface hypoplasia, down-slanting palpebral fissure, short nose, high palate, tented lip) and her mental development agree with KS. From her other features the cardiac anomaly is presented in 50 % of Kleefstra’s cohort, while in another review with nine patients a cardiac anomaly was observed in 87 % of the patients [3]. Another important symptom of our Patient 1 is the hearing loss, which was presented in 20 % of Kleefstra’s patients and is not reported in any patient of Iwakoshi’s cohort. From 22 months of age she had epileptic seizures well-controlled with AED. This symptom was presented in similar rate in Kleefstra’s (30 %) and in Iwakoshi’s (50 %) group, conversely hypotonia was presented in all of Iwakoshi’s and in 50 % of Kleefstra’s patients.

Similar to Patient 1 the phenotype and mental development of our Patient 2 are also characteristic for KS and the diagnosis was confirmed by FISH examination. The symptoms of Patient 2 are also evaluated (Table 1). Behavioral problems which are slightly more common in patients with intragenic mutations as in patients with 9q34.3 deletion could be observed in Patient 2 at the age of 3 years [9]. He had a known, but rare symptom of KS, namely the tracheomalacia, making him susceptible to pneumonia in his first years of life. At 20 month of age, first, focal epileptic seizures presented, which became, after a short therapy responsive period, therapy resistant. Meanwhile an abnormal AED metabolism became evident. This feature has not been published as part of KS and there is no data to be found in the literature that the haploinsufficiency of *EHMT1* causes a symptom as seen in our patient [10].

**Fig. 3:**
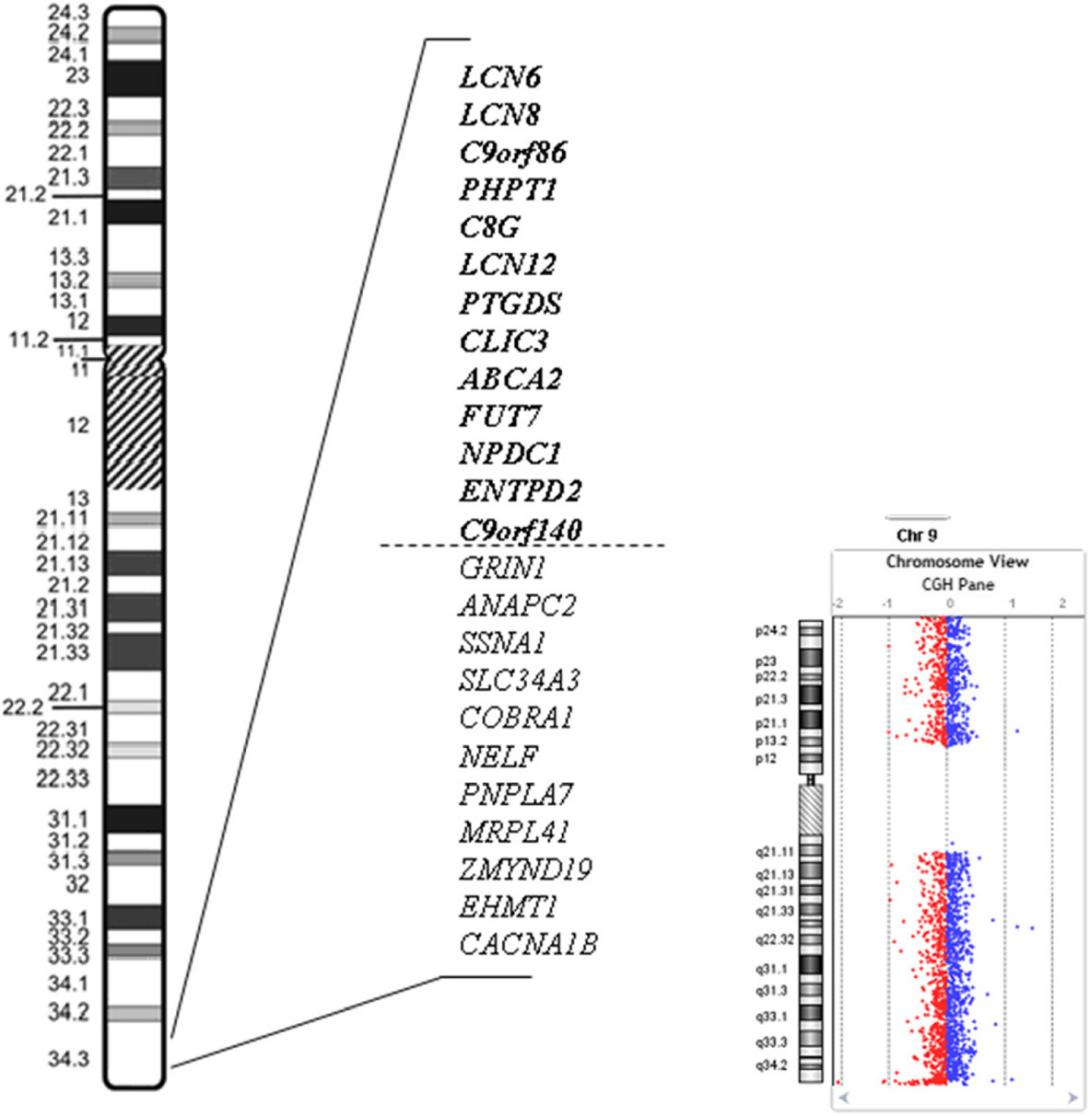
Demonstration of genes located in the 1.211 Mb deleted region in 9q34.3 (ch9:139,641,471–140,852,911) of Patient 2

As the result of the completed aCGH a 1.211 Mb deletion with the breakpoints ch9:139,641,471–140,852,911 was detected (the deleted region is represented by probes A_14_P200264 → A_16_P38909067). In their study Yatsenko et al. defined a minimal critical region of ~700 kb which was deleted in all of the patients they studied [6]. They suggest that the genes located in this region are responsible for the common clinical features and occurrence of other features depends on the size of the deletions. In another study of these authors deletions of 28 patients are interpreted, except of one all patients’ phenotypes fit with the syndrome [11]. In the phenotype of our patient the most of the symptoms agree with KS, except the abnormal AED metabolism. On a figure the genes located in the deletion of our Patient 2 are demonstrated (Fig. 3). In this region there are some genes which may have a potential effect on AED metabolism or impact. The proteins of these genes are expressed in brain and may influence and limit the penetration, efficacy, transport and bioavailability of the administered drugs via altered channel function or neural impairment. The mechanism of action of levetiracetam is unknown, but it is thought, that it stimulates synaptic vesicle protein 2A (SV2A) and inhibiting neurotransmitter release [12]. As regards to clonazepam, this benzodiazepine AED also displays inhibitory properties through the gamma-aminobutyric acid (GABA) receptor, a ligand-gated chloride ion channel, activated by GABA [13]. One of the mentioned genes, the *ABCA2* gene encodes a membrane-associated protein by this gene is an ABC-transporter family member that is highly expressed in brain tissue and we supposed that it may have a role in neural development [14]. The *ABCA2* is important in the central nervous system and in lipid transport necessary for the myelination process [15–17]. Previous findings of other researchers suggest that *ABCA2* is a novel lysosome-associated membrane protein involved in myelinization or other kinds of metabolism in the central nervous system [15]. Two other genes in this region are channel genes. The *PHPT1* gene encodes an enzyme that catalyzes the dephosphorylation of histidine residues in protein, and in this way may be part of the group of the calcium-activated potassium channel genes [18]. Many human hereditary diseases are associated with abnormal phosphorylation of cellular proteins [19]. The other one is the *CLIC3* gene, which encodes a p64 family member protein that stimulates chloride ion channel activity [20]. Chloride channels regulate fundamental cellular processes including stabilization of cell membrane potential, transepithelial transport, maintenance of intracellular pH, and regulation of cell volume and various chloride channels have also been implicated in human diseases [21–23]. Also an interesting point is the *C9orf86* (chromosome 9 open reading frame 86, also known *as RABL6*) gene, the encoded protein plays a role in neurotransmitter release in the membrane of synaptic vesicles and the *NPDC1* gene with a role in neural proliferation, differentiation and control [24, 25].

Because of ambiguous assumptions, we carried out the aCGH analysis in case of Patient 1 as well, to find the probable responsible gene in the background of the abnormal AED response. Interestingly, after aCGH examination, the observed deletion in the subtelomeric region of chromosome 9q was larger in Patient 1 compared to the deletion was found in Patient 2 (2.188 Mb vs. 1.211 Mb, respectively). Twenty-four additional genes (*UBAC1, LHX3, QSOX2, CARD9, PMPCA, INPP5E, SEC16A, NOTCH1, MIR126, AGPAT2, LCN10, NACC2, C9orf69, LOC26102, GPSM1, DNLZ, SNAPC4, SDCCAG3, C9orf163, EGFL7, FAM69B, SNHG7, SNORA4* and *SNORA17*) were detected in Patient 1 beyond the deleted genes in Patient 2, which also contained the *EHMT1* gene responsible for KS. Subsequently, the above mentioned genes presumably have no role in the insufficient drug metabolism and effect, and our earlier conjecture was disproved. Further clinical and laboratory investigations are needed to identify the causes behind the modified AED response, which presumably could not be explained with chromosomal abnormalities, rather with DNA sequence alterations or modified epigenetic mechanisms.

In conclusion, we report two Hungarian KS patients, one with the classic phenotype of the syndrome, while in our other patient beside the classic phenotype a new symptom – namely an abnormal drug metabolic response – could be observed.

### Materials and methods

Because of the abnormal drug metabolism, we performed aCGH analysis as well in Patient 2 searching for other rearrangements. Chromosomal microarray indicated only a large – 1.211 Mb -, deletion in the 9q subtelomeric region with the breakpoints ch9:139,641,471–140,852,911. No other CNV was detected in the index patient. Parental samples were analyzed using the same array and gave normal esults. The detailed data of the detected deletion is depicted on Fig. 3.

Subtelomeric FISH was performed on metaphase chromosomes prepared from peripheral blood according to the protocol of the manufacturer of the probe used (Vysis ToTelVysion Multicolor FISH Probe Kit, Abbott Molecular Inc., USA). The specimens were evaluated in epifluorescent microscope (Zeiss, Axioskop 2). Visualization and analysis was done using CytoVysion software (Applied Imaging, UK).

Array CGH was performed using the Agilent Human Genome G3 Sureprint 8x60K Microarray (Agilent Technologies, USA), a high resolution 60-mer oligonucleotide based microarray containing 55,077 60-mer probes, spanning coding and non-coding genomic sequences with median spacing of 33 kb and 41 kb, respectively.

Pooled genomic DNA from peripheral blood leukocytes of phenotypically normal males or females from Promega was used as a reference (Promega Male/Female Reference DNA, Cat. No.: G1471 and G1521, Promega Corporation USA).

Labeling and hybridization were carried out based on the Agilent protocol (Agilent Oligonucleotide Array-Based CGH for Genomic DNA Analysis – Enzymatic Labeling Protocol v7.2; July 2012). Washing was performed according to the Agilent Protocol v7.2. Array image was acquired using an Agilent laser scanner G2565CA (Agilent Technologies, California, USA) and analyzed with the Agilent Feature Extraction software (v10.10.1.1.). Results were presented by Agilent Cytogenomics software (v2.9.2.4). DNA sequence information refers to the public UCSC database (Human Genome Browser, Feb 2009 Assembly; GRCh37:hg19).

The deletion detected was aligned to known aberrations listed in publicly available databases, such as the DECIPHER (Database of Chromosomal Imbalance and Phenotype in Humans using Ensembl Resources), DGV (Database of Genomic Variants), Ensembl and ECARUCA (European Cytogeneticists Association Register of Unbalanced Chromosome Aberrations).

### Consent

Written informed consent was obtained from the patient for publication of this Case report and any accompanying images. A copy of the written consent is available for review by the Editor-in-Chief of this journal.

## Abbreviations

aCGH: array comparative genomic hybridization
AED: antiepileptic drug
CNV: copy number variation
DECIPHER: database of chromosomal imbalance and phenotype in humans using ensembl resources
DGV: database of genomic variants
ECARUCA: European Cytogeneticists Association Register of Unbalanced Chromosome Aberrations
FISH: fluorescent in situ hybridization
KS: Kleefstra syndrome
MRI: magnetic resonance image
OFC: occipitofrontal circumference

## References

[1] Kleefstrasyndrome Leaflet “Unique”. 2009. http://www.rarechromo.org/information/Chromosome%20%209/Kleefstra%20Syndrome%20FTNP.pdf.

[2] Kleefstra T, van Zelst-Stams WA, Nillesen WM, Cormier-Daire V, Houge G, Foulds N (2009). Further clinical and molecular delineation of the 9q subtelomeric deletion syndrome supports a major contribution of EHMT1 haploinsufficiency to the core phenotype. J Med Genet.

[3] Iwakoshi M, Okamoto N, Harada N, Nakamura T, Yamamori S, Fujita H (2004). 9q34.3 deletion syndrome in three unrelated children. Am J Med Genet A.

[4] Verhoeven WM, Egger JI, Vermeulen K, van de Warrenburg BP, Kleefstra T. Kleefstra syndrome in three adult patients: further delineation of the behavioral and neurological phenotype shows aspects of a neurodegenerative course. Am J Med Genet A. 2011/09/13 ed2011. p. 2409–15. 10.1002/ajmg.a.34186 21910222

[5] Kleefstra T, Smidt M, Banning MJ, Oudakker AR, Van Esch H, de Brouwer AP (2005). Disruption of the gene Euchromatin Histone Methyl Transferase1 (Eu-HMTase1) is associated with the 9q34 subtelomeric deletion syndrome. J Med Genet.

[6] Yatsenko SA, Cheung SW, Scott DA, Nowaczyk MJ, Tarnopolsky M, Naidu S (2005). Deletion 9q34.3 syndrome: genotype-phenotype correlations and an extended deletion in a patient with features of Opitz C trigonocephaly. J Med Genet.

[7] Kleefstra T, Kramer JM, Neveling K, Willemsen MH, Koemans TS, Vissers LE (2012). Disruption of an EHMT1-associated chromatin-modification module causes intellectual disability. Am J Hum Genet.

[8] Kleefstra T, Brunner HG, Amiel J, Oudakker AR, Nillesen WM, Magee A (2006). Loss-of-function mutations in euchromatin histone methyl transferase 1 (EHMT1) cause the 9q34 subtelomeric deletion syndrome. Am J Hum Genet.

[9] Willemsen MH, Vulto-van Silfhout AT, Nillesen WM, Wissink-Lindhout WM, van Bokhoven H, Philip N (2011). Update on Kleefstra Syndrome. Mol Syndromol.

[10] Balemans MC, Ansar M, Oudakker AR, van Caam AP, Bakker B, Vitters EL (2014). Reduced Euchromatin histone methyltransferase 1 causes developmental delay, hypotonia, and cranial abnormalities associated with increased bone gene expression in Kleefstra syndrome mice. Dev Biol.

[11] Yatsenko SA, Brundage EK, Roney EK, Cheung SW, Chinault AC, Lupski JR (2009). Molecular mechanisms for subtelomeric rearrangements associated with the 9q34.3 microdeletion syndrome. Hum Mol Genet.

[12] Wright C, Downing J, Mungall D, Khan O, Williams A, Fonkem E (2013). Clinical pharmacology and pharmacokinetics of levetiracetam. Front Neurol..

[13] Nutt DJ, Malizia AL (2001). New insights into the role of the GABA(A)-benzodiazepine receptor in psychiatric disorder. Br J Psychiatry..

[14] Mack JT, Brown CB, Tew KD (2008). ABCA2 as a therapeutic target in cancer and nervous system disorders. Expert Opin Ther Targets.

[15] Zhou C, Zhao L, Inagaki N, Guan J, Nakajo S, Hirabayashi T (2001). Atp-binding cassette transporter ABC2/ABCA2 in the rat brain: a novel mammalian lysosome-associated membrane protein and a specific marker for oligodendrocytes but not for myelin sheaths. J Neurosci.

[16] Schmitz G, Kaminski WE (2002). ABCA2: a candidate regulator of neural transmembrane lipid transport. Cell Mol Life Sci.

[17] Tanaka Y, Yamada K, Zhou CJ, Ban N, Shioda S, Inagaki N (2003). Temporal and spatial profiles of ABCA2-expressing oligodendrocytes in the developing rat brain. J Comp Neurol.

[18] Zhang XQ, Sundh UB, Jansson L, Zetterqvist O, Ek P (2009). Immunohistochemical localization of phosphohistidine phosphatase PHPT1 in mouse and human tissues. Ups J Med Sci.

[19] Hunter T (2000). Signaling--2000 and beyond. Cell.

[20] Qian Z, Okuhara D, Abe MK, Rosner MR (1999). Molecular cloning and characterization of a mitogen-activated protein kinase-associated intracellular chloride channel. J Biol Chem.

[21] Bretag AH (1987). Muscle chloride channels. Physiol Rev.

[22] Koch MC, Steinmeyer K, Lorenz C, Ricker K, Wolf F, Otto M (1992). The skeletal muscle chloride channel in dominant and recessive human myotonia. Science.

[23] Riordan JR, Rommens JM, Kerem B, Alon N, Rozmahel R, Grzelczak Z (1989). Identification of the cystic fibrosis gene: cloning and characterization of complementary DNA. Science.

[24] Ádám V (2001). Orvosi biokémia. 1st ed. A kémiai idegingerület átvitel (neurotranszmisszió) molekuláris alapjai.

[25] Dupont E, Sansal I, Toru D, Evrard C, Rouget P (1997). Identification of NPDC-1, gene involved in the control of proliferation and differentiation of neural and glial precursors. C R Seances Soc Biol Fil.

